# Maximum performance, repeatability, and intraindividual variability of sprinting in common wall lizards ( *Podarcis muralis* )

**DOI:** 10.17912/micropub.biology.002072

**Published:** 2026-09-14

**Authors:** Madelyn C Browning, Lindsey Mayor, Halle Kozak, Gabrielle Abshire, Isabel Allo, Alida C Askill, Allison Bogan, Hayley Browning, Halle Dacek, Lan Do, Sam Frary, Sydney Gassert, Aida Gesink, Caleb Gunn, Landon Key, Ella M Neuenschwander, Brittney C Parks, Evelyn Waltz, Aidan Weir, Eric J Gangloff, Bryan H Juarez

**Affiliations:** 1 Ohio Wesleyan University, Delaware, OH, US

## Abstract

The repeatability of functional traits like physiological maxima (maximum performance) measures the reliability of underlying measurements. However, best practices for analyzing maximal performance while accounting for within-individual variation are lacking. Here, we quantify the coefficient of variation and repeatability of maximum sprinting speed in common wall lizards ( *Podarcis muralis* ) from Cincinnati, USA. We use insights from experimental data and bootstrap resampling to discuss strategies for analyzing physiological maxima while accounting for within-individual variation. Relative to assuming greater among- than within-individual variation, our proposals offer greater precision in parameter estimates and statistical test results with increased power, thus improving scientific reproducibility.

**Figure 1. The effect of intraindividual variation on the repeatability of maximum sprinting speed in common wall lizards ( Podarcis muralis ) from Cincinnati, Ohio, USA.** A. The distribution of maximum sprinting speeds across lizards ordered by mean. The small female sprinted at an average of 0.37 m/s (standard error (SE) = 0.013 m/s), small male 2 at 0.39 m/s (SE = 0.018 m/s), large male at 0.46 m/s (SE = 0.0088 m/s), large female at 0.47 m/s (SE = 0.012 m/s), and small male 1 at 0.52 m/s (SE = 0.019 m/s). B. The percent of top speed sampled by the maximum of N measurements in the two lizards with the most dissimilar patterns. Error bars are the 25th and 75th sampled quantiles. The formula is the global population-level average effect. C. The repeatability of maximum sprinting speed based on N-sample averages. Error bars are the 25th and 75th sampled quantiles. The formula is the global population-level average effect.

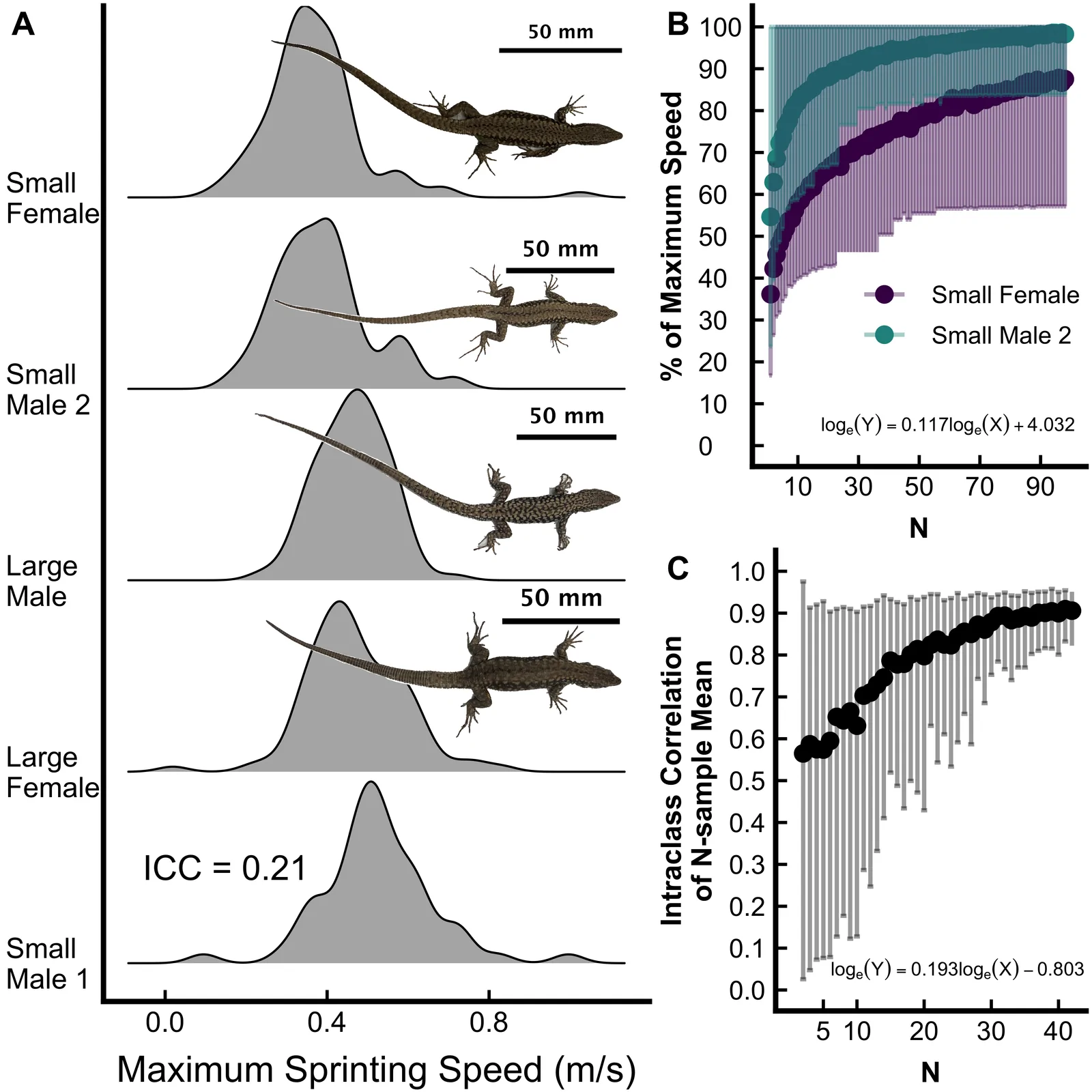



## Description

Reproducibility is a tenet of the scientific method; however, natural variability can compromise reproducibility. For example, patterns within species may not be easily reproduced due to intraspecific variability emerging from random variation among individuals or repeated measurements on the same individuals that may change through time (e.g., due to behavioral or physiological change). Although researchers often analyze maximum values without accounting for within-individual variation (by assuming relatively greater among-individual variation), the risk of doing so is great: the study is not reproducible if the assumption is falsifiable. The purpose of this study is to measure among- and within-individual variability to determine the extent to which experiments on maximum sprinting speed in *Podarcis muralis* can be expected to be reproducible.

Organismal performance, an individual’s ability in a specific task, bears important implications for individual fitness, ecology, and evolution (Arnold 1983; Husak et al., 2006; Irschick and Meyers 2007). Some examples include measuring whole-animal movement, bite force, or contractile velocities in muscles (Garland et al., 1990; James et al., 2007; Anderson et al., 2008). Most performance studies analyze an individual’s maximum performance, but this is often done using low sample sizes associated with relatively larger errors (Losos et al., 2002; Adolph and Pickering 2008). In this study, we assess the expected scientific reproducibility of maximum sprinting speed by quantifying the relationships between maximum sprinting speed, repeatability ( *sensu* Falconer and Mackay 1996), intraindividual variability, and sample size in introduced common wall lizards ( *Podarcis muralis* ) from Cincinnati, Ohio, USA. In our experience, different individuals often exhibit different maximum sprinting speeds and the same individuals tend to run at similar speeds from day to day (Gangloff et al., 2019). Therefore, we expect maximum sprinting speed to be repeatable and studies of maximum sprinting speed in this species to be scientifically reproducible, although large sample sizes may be required to accurately estimate per-individual maxima.

We measured the repeatability of sprinting speed among five lizards totalling N = 387 trials. The individuals selected for this study were chosen to represent sex- and size-specific variation found in these introduced populations. These five lizards included one smaller male (body mass = 5.13 g, snout-vent length = 60.30 mm, sprinted N = 56 times) which died during the study period and was replaced with a second smaller male (4.53 g, 55.54 mm, N = 42), a larger male (7.26 g, 64.28 mm, N = 96), a smaller female (5.17 g, 58.70 mm, N = 95), and a larger female (7.48 g, 66.00 mm, N = 98). We measured mass across the trial period and found no change (GLMM; Slope = -0.012, t = -0.325, DF = 320, p = 0.745). The lizards sprinted an average of 0.44 m/s ( Fig. 1A ) and this did not change throughout the study (LMM; Slope = -0.0279, t = -3.752, DF = 1.0354, p = 0.159). The coefficient of variation (CV) for sprinting speed across individuals ranged from 0.19 to 0.33 with an average of 0.27. We found sprinting speed was weakly repeatable (ICC = 0.21): only 21% of variation in sprinting speed was due to among-individual variation. As predicted, it is also challenging to estimate the per-individual sampled maximum as large sample sizes are required ( Fig. 1B ). For example, N = 11 and 55 trials guarantee sampling a maximum speed of 75 and 90% of the animal’s top speed. Previous studies have described difficulties in estimating an animal’s true maximum (Adolph and Pickering 2008; Astley et al., 2013). Additionally, we found a logarithmic relationship between sample size and the repeatability of maximum sprinting speed across the average of N samples ( Fig. 1C ). We discuss methods for overcoming limited repeatability and high sample size requirements to improve scientific reproducibility below.

*P. muralis* exhibited greater intraindividual variability relative to similar species. Since sprinting speeds in ectotherms are temperature-dependent, published studies report a coefficient of variation across groups of interest of 0.05–0.32, for *P. muralis* at 22°C (Gomes et al., 2017; Žagar et al., 2017). In N = 21 adult and subadult male *Sceloporus occidentalis* (western fence lizards), mean CV across individuals was 0.20–0.23 at 20 and 35°C (Adolph and Pickering 2008). These findings indicate that the CV of maximum sprinting speed may be greater in *P. muralis* than in *S. occidentalis* , but the differences in the measured temperatures and number of trials and lizards may confound comparisons and further work is needed. Furthermore, a previous study reports a higher sprinting repeatability (constancy) of 0.5 for *P. muralis* measured at 31°C using N = 82 lizards (Gangloff et al., 2019). These findings suggest the repeatability of maximum sprinting speed may be lower in *P. muralis* from the USA, relative to Europe, but further research is needed. In *S. occidentalis* , sprinting repeatability was 0.31 and 0.83 for juvenile populations measured at 24 and 30°C, respectively (Buckley et al., 2009). Maximum sprinting speed was repeatable (0.56–0.63) across years in N = 30–62 adult male *S. merriami* (Huey and Dunham 1987). Overall, maximum sprinting speed in *P. muralis* exhibited a low repeatability despite having a low CV.

Here, we describe alternative methods for accounting for within-individual variation. Although we often assume greater among- than within-individual variation (ICC > 0.5), these assumptions are not explored often in the broader biological literature. We emphasize that quantifying repeatability in maximum performance traits is an important first step in understanding the ecology and evolution of physiological performance (Bennet and Huey 1990; Roche et al., 2016). This is partly because any degree of within-individual variation (ICC < 1) results in relatively larger variance of parameter estimates and lower statistical power. One way to overcome these issues is to use random effects models to jointly estimate and distinguish among- and within-individual variation (Gelman and Hill 2007). A second method is to average over individual variability by estimating per-individual means of maximum performance using N ≥ 2 samples; for *P. muralis* , measuring each individual N = 2 or 10 times results in repeatabilities of about 0.5 and 0.7, respectively ( Fig. 1C ). Notably, applying non-normal residual distributions or using permutational methods (Collyer and Adams 2018; 2024) may be necessary when N < 30 and speed was not measured using instantaneous measurements (e.g., with high-speed video) because (“interval”) estimates from infrared sensors are not normally distributed and have relatively greater variance (Gomes et al., 2017). These approaches have the inherent benefits of controlling for behavioral, physiological, or environmental effects on intraindividual variability (Losos et al., 2002; Roche et al., 2016), including plasticity (Irschick and Meyers 2007; Stamps 2016). Determining appropriate sample sizes remains largely study-specific and it is up to researchers to determine appropriate trade-offs between repeatability (Liljequist et al., 2019), statistical power (Serdar et al., 2021), researcher effort, and animal ethics. In conclusion, we have quantified the relationships between repeatability, intraindividual variation, and sample size for sprinting speed in common wall lizards and used these insights to describe a statistical framework for analyzing maximum performance data and making appropriate conclusions in a scientifically reproducible manner.

## Methods

We caught lizards by hand or using the lasso method (Blomberg and Shine 2006) from two sites (Ault and Mount Storm Parks) in Cincinnati, Ohio, USA during May 2025. Animal care was identical to a previous study (Head et al., 2024), except we housed each female with 1–4 other non-experimental females and housed males in pairs with non-experimental males or singly in plastic tubs (~43 cm L × 31 cm W × 33 cm H). We conducted all research under Ohio Division of Wildlife Wild Animal Permit (23-014) and all procedures were approved by Ohio Wesleyan University Institutional Animal Care and Use Committee (2020-21-04).

We measured sprinting speed using published methods (Vaughn et al., 2021; Head et al., 2024), except we used a 1-m racetrack with a paving stone substrate. We initially chose two male and female pairs of lizards for sampling which had complete tails and represented the greatest difference in body size in our animal colony. The animals spent 50–75 minutes in plastic containers in an incubator at 34°C before trials. Preliminary trials with non-experimental animals showed this procedure got animals within ±0.5°C of their optimal temperature for sprinting, which was ~33°C for the sampled populations (unpublished). Animals were measured 2–4 times during trials occurring once or twice per day, between 0900 and 1530 hours, for 14 days from 28 October 2025 to 30 November 2025. On days with two trial periods, we rested the animals at room temperature (~20°C) for 1 hour between trials. We measured maximum speed as the animal’s fastest 25-cm dash. We measured mass during most trial periods using a Top-200 scale (Weigh Gram, Barcelona, Spain) and snout-vent length (SVL) before or immediately after the experimental period using a CD-6 ASX digital caliper (Kawasaki, Kanagawa, Japan). 

We investigated repeatability and individual variability in a number of ways. First, we used a generalized linear mixed model with random slopes and intercepts (for specimen ID) to test for changes in mass and velocity through the experimental period. The mass model included a first-order continuous autocorrelation structure to account for temporal autocorrelation. We implemented these models using the *nlme* v3.1-168 (Pinheiro and Bates 2000; Pinheiro et al., 2025) and *lme4* v1.1-37 (Bates et al., 2015) libraries in R v4.5.0 (R Core Team 2025); we estimated the DF and p-value for the lmer model fit using Satterthwaite's method via the *lmerTest* package v3.2-1 (Kuznetsova et al., 2017). Next, we estimated the individual and average coefficient of variation for sprinting speed. Then, we estimated the intraclass correlation coefficient (ICC) using the *psych* package v2.5.6 (Revelle 2025). Specifically, we estimated the ICC2 (Koo and Li 2016) single measurement (k=1) statistic representing absolute similarity (agreement) across samples. This statistic is the same as the ICCa statistic (Collyer and Adams 2018; 2024) and the ICC(2,1) statistic (Shrout and Fleiss 1979). The ICC ranges from 0 to 1; ICC = 1 represents high repeatability and a high ratio of among-individual to total variation. Additionally, we standardized velocities using lizard-specific maxima and used bootstrap resampling (1,000× with replacement) to determine the relationship between sample size and each animal’s sampled speed, relative to individual maxima. Finally, we used a similar resampling approach to quantify the repeatability of average maximum sprint performance (the ICC2k statistic) across N samples per individual, up to the minimum individual sample size (N = 42).
